# Equine Transport-Related Problem Behaviors and Injuries: A Survey of Italian Horse Industry Members

**DOI:** 10.3390/ani11010223

**Published:** 2021-01-18

**Authors:** Francesca Dai, Martina Zappaterra, Michela Minero, Francesca Bocchini, Christopher B. Riley, Barbara Padalino

**Affiliations:** 1 Dipartimento di Medicina Veterinaria, Università degli Studi di Milano, via dell’Università 6, Lodi, 20122 Milano, Italy; francesca.dai@unimi.it (F.D.); michela.minero@unimi.it (M.M.); 2 Dipartimento di Scienze e Tecnologie Agro-Alimentari, Alma Mater Studiorum—Università di Bologna, Viale Fanin 50, 40126 Bologna, Italy; martina.zappaterra2@unibo.it (M.Z.); francesca.bocchini2@studio.unibo.it (F.B.); 3 School of Veterinary Science, Massey University, Palmerston North 4470, New Zealand; c.b.riley@massey.ac.nz

**Keywords:** horse, transport, injury, behavior, welfare

## Abstract

**Simple Summary:**

The aim of this study was to identify risk factors for equine transport-related problem behaviors (TRPBs) and injuries in support of the development of best practices that minimize their occurrence. An online cross-sectional survey was designed and disseminated to equine industry members in Italy. Respondents were asked if one of their horses exhibited TRPBs or sustained transport injuries during the two previous years, and to describe their equine background, experience, transport vehicles, and practices. TRPBs were reported by 14.45% of respondents. Sedation and coercive equipment (i.e., whip) use for loading were confirmed to be major risk factors for horse injuries (17/148; 11.49%). Horse injuries were also associated with a lack of checks of the vehicle brakes, and vehicle design (i.e., rubber mat and chest bar). During 50% of the accidents described, horses and handlers were simultaneously injured. These results may be useful to safeguard horse and handler well-being by educating people in charge of moving horses on transport risks and best practices.

**Abstract:**

An online survey was conducted to determine associations between equine transport management and transport-related injuries and problem behaviors in Italy. The survey was composed of four sections: respondents’ demographic information and background, transport management practices, journey details and vehicle design, and transport injuries experienced by the horse in the previous two-year period. Univariable and multivariable logistic regression with a binary outcome variable was performed to explore associations between variables (respondents’ and journeys’ details and transport practices) and equine transport-related problem behaviors (TRPBs) and injuries. TRPBs were also considered an explanatory variable for injuries. The survey generated 201 responses; only 148 were complete and analyzed. TRPBs were reported by 14.45% of the respondents and the odds of TRPBs was linked to the respondent gender (*p* = 0.034), the use of tranquilizers prior to transport (*p* = 0.002), the use of a whip for loading (*p* = 0.049), the lack of protection equipment (*p* = 0.050), and shavings (*p* = 0.025) on the vehicle floor. Horse injuries (11.49%) were reported by more respondents who did not check the brakes of their transport vehicle before traveling (*p* = 0.043), had vehicles with padding on the chest bar (*p* = 0.038), and for horses reported to display TRPBs (*p* = 0.001). Finally, 10 respondents reported they were injured during horse transport (10/140; 7.14%), 50% simultaneously with their horses. The study findings should be interpreted with caution due to small sample size bias and participants’ recall bias. Nevertheless, the results are in concordance with the literature, confirming that horse transport is a risk for the horse’s and handler’s health and well-being. Further studies are needed to identify best management practices to educate equine industry members on how to minimize transport-related problems.

## 1. Introduction

Second only to trauma sustained in the paddock or yard, injuries to horses in the transport vehicle occur frequently [1]. At loading, limb injuries associated with the loading ramp are common. During the journey, halter rubbing at the poll or muzzle and tail rubbing are specific abrasion types that may occur. Wounds to the withers are caused by contact with the vehicle ceiling, whereas leg wounds in transit most commonly occur due to loss of balance after braking and cornering. Rapid and extreme braking can result in more severe injuries such as vertebral fractures and joint dislocation in horses facing forward and restrained with short tie-ropes [2]. The incidence of transport-related horse injuries varies from 1.6% to 33% depending upon the population studied, and most investigations have been of horses for human consumption transported by road using commercial companies [3,4,5]. In Australia, injuries associated with commercial and non-commercial equine transport were reported by 45% of surveyed respondents within two years before survey completion [6]. In a Swedish survey, 12% of equestrian horse owners reported an equine injury during loading [7]. A face-to-face survey conducted at equestrian events in Southern Australia focused on non-commercial horse transport and found that 25% of the respondents had experienced a transport-related injury within the 15 years prior to the study [8]. In New Zealand, 17.7% of horses are at risk of injury during transport, most frequently sustained while in transit (70%) [9]. Identified risk factors for horse injury during transport are human factors, the choice to use protective equipment on the horse and administration of sedation [6], breed, journey duration, failing to perform a mechanical checklist prior to transport, transport-related problem behaviors (TRPBs), the travel experience of the horse, using a tail guard, and the method of training horses for transport [10]. Human-related risk factors include the age of the respondents (younger versus older), type of involvement in the industry (amateur or professional), driver error, and telephone use while driving [6,8,9,10].

Transport-related problem behaviors (TRPBs) are defined as “any transport-related behavior that impedes welfare or safety of the horse or handler during the transportation process” [11]. These behaviors can occur during the pre-loading phase, due to separation from familiar environments [12], interactions with humans [13], and learned associations with past travel experiences [14]. In this phase, horses may exhibit signs of anxiety, e.g., vocalization, pawing, increased locomotion, and shaking [12]. Horses are most likely to exhibit TRPBs during loading in comparison to pre-loading, during or after travel [7,15]. They may show signs of anxiety when approaching the vehicle, due to innate phobia or aversion to confined spaces [16,17]. TRPBs in this phase include avoidance behaviors (e.g., rearing, pulling away sideways or backwards), or stress-related behaviors (e.g., pawing, kicking out, bolting, or head-shaking) [18,19,20,21]. During travel, TRPBs are generally exhibited during the first hour, due to the need of the horse to adapt to the vehicle and the motion [22,23]. Reported TRPBs in this phase are vocalization, head tossing, pawing, scrambling, head-turning, kicking out at the vehicle, biting and kicking directed at traveling companions, and reduced feeding/drinking [8,15,19,24,25,26]. Finally, TRPBs exhibited during unloading procedures include a reluctance to exit the vehicle or leaving the vehicle at excessive speed [17]. TRPBs have been associated with an increased risk of equine injury during transport [27]. A survey on horse road transport in Australia reported a high proportion (75%) of incidents associated with behaviors such as scrambling, slipping, and horse–horse interactions [8]. Similarly, a survey conducted in the UK attributed the cause of 55.6% of the incidents during transport to horse behavior [10]. TRPBs have been associated not only with horse injuries but also with human injuries. In a Swedish survey of equestrian horse owners, 5% of the respondents described concurrent injury of the animal being loaded and the handler [7].

In Italy, approximately 367,000 [28] registered horses are involved in sports (from amateur to professional level), leisure, and animal-assisted therapy. Horses not destined for food production are therefore transported for several reasons (e.g., competing, transfer to therapy centers). Despite the high number of horse movements in Italy, data on commercial and non-commercial transport issues and management are scarce. To fill this gap, the aim of the present work was to explore the prevalence and the risk factors for transport-related behavioral problems and injuries in horses occurring during transport in Italy from 2018 to 2020.

## 2. Materials and Methods

This online survey was approved by the Massey University Human Ethics Committee as low risk (Ethics Notification Number: 4000017178).

### 2.1. Respondents

The target population for this survey (see Supplementary Materials) was Italian residents with first-hand involvement in the road transport of one or more performance or recreational horses for professional or recreational purposes. To qualify for inclusion in the study, participants were required to have at least one horse in their care and to have been involved in at least one horse transport event during the two years prior to completing the survey. Respondents took part in the study voluntarily. In Italy, 367,561 horses were registered in 2017 [29] and often one owner/trainer was responsible for more than one horse. Based on an estimated target population of 125,000 equine industry participants [28], 383 surveys were required to attain a 95% confidence level and an error level of ±5% [30].

### 2.2. Survey

The survey was adapted from one used for previous studies by Padalino and colleagues [6,9,31,32]. The survey was translated into the Italian language to make it accessible to the target population (an English version of the survey is shown in the supplementary materials, SM1). Online proprietary software (Qualtrics, New Zealand) was used to build the survey and facilitate distribution. 

Invitations to contribute to the survey were shared via social networking sites (Table 1). Italian horse-related web pages were contacted to invite them to share the survey link. Italian horse organizations were also contacted by email requesting them to publish the link to the survey on their webpage. The personal webpages of the authors also provided access to the survey. The survey link was available for completion for 3 months, from January to March 2020.

The survey consisted of 37 closed and 5 open-ended questions. The first part of the survey explored human-related factors such as demographic details (gender, age, origin) of the respondents, information on their involvement with the equine industry, the nature of their involvement with horses (professional or amateur), their experience with horses, education and training, class of driving license, understanding of the health and safety at work act 81/2008, understanding of Regulation 1/2005/CEE on live animal transport, and their ability to recognize equine distress. The second part of the survey explored horse and journey details and transport practices. The latter was split into respondents’ pre-transport practices, loading practices, and transport practices. Participants were then asked if their horses had shown a TRPB or sustained a transport-related injury during the two years prior to completing the survey (2018–2020), and to describe them. Finally, seven questions explored the occurrence of human injuries related to horse loading and transport (Table 2).

### 2.3. Explanatory Variables

Quantitative data not fulfilling the requirements for parametric analyses (e.g., age, experience, number of horses, average journey distance) were transformed into categorical variables for further analysis using the approximate values of the 25th, 50th, or 75th percentiles for division into categories. Based on the replies concerning the use of equipment or aids for loading the horses (Q23), a variable summarizing the number of types of equipment or aids used was created. Furthermore, a dichotomous variable consisting of “Aids” (at least one equipment or aid used) and “No aids” was created. The answers to questions addressing each respondent’s approach to training their horses for loading and traveling were classified based on the following training method categories, as previously reported [9]: habituation (H; e.g., “Foals follow the mother in and out a trailer many times”), self-loading (SL; e.g., “I taught my horse to self-load on command”), no training applied (NT), operant conditioning with a combination of negative reinforcement and positive punishment (R-P+; e.g., releasing pressure with bum ropes or other R- tools and use of whip on unwanted behavior), operant conditioning using positive reinforcement (R+; e.g., using carrots), and not specified (NS).

To avoid categories with insufficient numbers of positive observations, the categories showing frequencies <5% and less than 10 positive observations [33] were combined to avoid an unbalanced data structure for regression. Respondents geographically outside of Italy (2/148) were included in the missing data category and excluded from further analyses. Horse industry respondents involved in “racing”, “breeding”, and “farmers of horses for meat purpose” were considered together in the category “Other.” Affiliations with ENGEA (Ente Nazionale Guide Equestri Ambientali), trot and gallop racing (previously UNIRE association), and SEF (Scuola Equestre di Formazione) were grouped into “Other association.” Concerning the type of driving license, the respondents without one or with a provisional driving license were grouped into the “No license” category. Furthermore, the self-loading (SL) and positive reinforcement (R+) categories of the variable type of training for loading and traveling were clustered together into the R+SL category.

Dichotomous variables (Yes/No) were created for the replies concerning equine-related qualifications (i.e., a training license), a mechanical check of the transport vehicle before transport (e.g., brakes, lights, tire pressure, wheel nuts, hydraulic fluid levels, sides/walls, floor, towbar attachment, windows, ventilation), and the use of sedation, protective equipment on the horse (e.g., boots, leg bandages, tail guard/bandage, body rug), aids to load the horse (e.g., whip, food), food en route, and the type of bedding used in the vehicle (e.g., straw, chips, shavings, rubber mat). Only six respondents replied that they used the poll protector for transport protection (frequency <5%); this variable was not further considered.

Table 2 shows the explanatory variables and their categories.

### 2.4. Outcome Variables

Respondents were asked if their horses had shown behavioral problems at the time of pre-loading, loading, transport, or unloading, such as fear/anxiety, refusal to load, flight responses, kicking, or scrambling. They were also asked if their horses had experienced any injuries (Q30) during transport in the last two years and if they had sustained a transport associated injury (Q35) within the same time period. Respondents were also asked to report the type of horse or horse handler injury (shallow or deep cut or wound, fracture or broken bone, bruise, or others, as described by the respondents) and in which phase of transport (pre-loading, loading, traveling, or unloading) it happened. The respondents also provided information concerning the location of the injury on the body of the horse or horse handler and the recovery time.

The dichotomous variables (presence/absence; yes/no) of TRPBs and transport-related injuries in horses were considered outcome variables in regression models.

### 2.5. Statistical Analyses

Data were downloaded from Qualtrics in an Excel file format and organized as previously mentioned. Descriptive statistics of all predictive variables, identified as categorical, were performed using the Statulator^®^ online free software [34] and reported as counts and percentages. The dichotomous variables of TRBPs and horse injuries were used as outcomes for univariable logistic regression models, and the variables in Table 2 were used as predictive variables. Additionally, the presence/absence of TRBPs was also considered among the predictive variables for the presence/absence of transport-related horse injuries. The results were reported as an odds ratio (OR), confidence interval 95% (CI 95%), and *p*-values. The *p*-values of each predictive variable tested in univariable logistic regression were calculated using the Wald test, and for each outcome, the variables that showed a *p*-value < 0.25 were considered for inclusion in backward stepwise multivariable logistic regression models. The backward elimination was run manually. Observations with missing values were automatically excluded from the analyses. Predictive variables were removed until all variables in the final model had a *p*-value < 0.15 and the lowest Akaike information criterion (AIC) value for the model was attained. A *p*-value < 0.15 was set as threshold following the default value used in other statistics software. The results of the stepwise multivariable logistic regression models are presented as the odds ratio (OR), confidence interval (95% CI), and *p*-value for each predictive variable.

The male gender of the respondents correlated with high knowledge of the health and safety at work act and the absence of TRBPs (i.e., the respondents who claimed no behavioral problems in their horses and answered “high” to the question about their knowledge of the health and safety at work act were all males). Thus, gender was considered in the stepwise multivariable logistic regression model for TRBPs. The variables “whip” and “other aids” used during loading were collinear, and only the “whip” variable was tested in subsequent stepwise multivariable logistic regression models. The inclusion of these variables in the stepwise multivariable logistic regression model was tested based on their capability to explain the model variability, and the model relative quality criterion (Akaike information criterion—AIC).

The scripts used to perform the univariable logistic and stepwise multivariable logistic regressions were a combination of functions in the packages *nlme* [35], *lsmeans* [36], *lme4* [37], and *car* [38] in an R environment [39].

## 3. Results

### 3.1. Survey Response

A total of 210 people responded to the questionnaire. Among them, 148 (70.5%) responded to the questions concerning whether their horses showed TRPBs and whether the horses or horse handlers experienced transport-related injuries in the previous two years, and thus were further considered for the subsequent data analyses. The number of respondents resulted in an 8% error rate at the 95% confidence level and did not reach the survey target sample size.

### 3.2. Descriptive Statistics

#### 3.2.1. Descriptive Statistics of the Categorical Variables

Most respondents were female (111/144; 77.08%). Half were 18 to 30 years old (73/145; 50.34%). Twenty-two respondents did not answer the question concerning their geographic location within Italy (22/148). Among those that answered this question (126), most were from northern Italy (91/126; 72.22%), 20 were from central Italy (20/126; 15.87%), and 15 from southern Italy (15/126; 11.90%) (Table S1).

There were 77 respondents (77/148; 52.03%) who handled horses involved in equestrian sports (ES), 35 in recreational riding (RR; 35/148; 23.64%), 19 in Western (W; 19/148; 12.84%), and 17 in the “Other” class (17/148; 11.49%). Most respondents were affiliated with an industry association (122/148; 82.43%), in particular, 89 with FISE (89/148; 60.14%), and most were involved as amateurs in the industry (97/147; 65.99%). The descriptive statistics for respondents’ details are reported in the supplementary materials (Table S2).

Descriptive statistics of results for the questions about the experience/knowledge of the respondents are given in Table 3. Among the respondents that replied (140/148), the majority had more than 10 years of experience but only had a driving license for cars (type B). Concerning the questions about the self-assessment of knowledge of the regulations concerning health and safety at work and animal welfare during transport, there was a balance of frequencies between the classes. One respondent (1/148) did not reply to the question about the self-assessment of the ability to identify a horse in distress; most of the 147 replied that they had a moderate or high ability.

Table 4 reports the descriptive statistics for horse and journey details. Among the respondents that indicated the number of horses they were responsible for, there was a balance of frequencies between the classes. Most respondents did not transport their horses frequently and assessed the horses’ fitness for travel before transport about half of the time.

Table 5 reports the descriptive statistics for the questions about pre-transport practices. Six respondents did not indicate how often they performed a mechanical checklist, and eight did not indicate which mechanical parts they checked. Of those that responded, one third indicated they never performed mechanical checks. The mechanical parts checked most frequently were brakes, lights, tire pressure, towbar attachment, and ventilation. About 11% of the respondents (17/146; 11.64%) used sedatives or other products to calm the horse before transport.

Among the responses to the questions concerning equipment for protection during transport (146/148), 27 indicated they did not use protective equipment (27/146; 18.49%), 27 used one type (27/146; 18.49%), 49 used two (49/146; 33.56%), 30 used three (30/146; 20.55%), and 13 replied that they used four or more protective devices (13/146; 8.90%). The most frequently used protective equipment was leg bandages (94/146; 64.38%) and tail guards/bandages (82/146; 56.16%). Furthermore, among the respondents, 60 used a body rug (60/146; 41.10%) and 31 used leg boots (31/146; 21.23%). Most respondents trained their horses for loading and traveling (92/148; 62.16%). Concerning the type of training used, 34 did not specify it (NS; 34/148; 22.97%), 25 replied that they used habituation (H; 25/148; 16.89%), 15 used positive reinforcement and self-loading (R+SL; 15/148; 10.14%), and 18 used negative reinforcement and positive punishment (R-P+; 18/148; 12.16%) (Table S3). 

Table 6 reports the descriptive statistics for the questions about loading practices. Forty-three respondents (43/148; 29.05%) replied that they used aids during loading and often coercive aids such as bum ropes and whips.

For containment within the vehicle (147/148), most used cross ties (75/147; 51.02%), 33 tied horses up with a short rope (33/147; 22.45%), 29 tied them with a long rope (29/147; 19.73%), and 10 used no restraint (10/147; 6.80%). Vehicles design features for horse protection were used by most of the respondents (133/148; 89.86%). These included padding on partitions (97/148; 65.54%), padding on the bum bar/behind the horse (85/148; 57.43%), padding on the chest bar (67/148; 45.27%), and partitions extending to the floor (29/148; 19.59%). Eighty-four replied that they provided food en route (84/148; 56.76%). Concerning bedding in the trailer, rubber mats were common (88/147; 60.27%), followed in descending order of frequency by shavings (86/147; 58.50%), straw (22/147; 14.97%), and sawdust (19/147; 12.93%) (Table S4). 

#### 3.2.2. Descriptive Statistics of the TRPBs

Twenty-one respondents (21/145; 14.45%) reported having at least one horse showing TRBPs (Table S5). Of these, seven (7/21; 33.33%) declared that horses showed fear and anxiety, seven (7/21; 33.33%) indicated that the animals refused to load into the vehicle, five (5/21; 23.82%) indicated that they noticed the horse kicking during transport, one (1/21; 4.76%) scrambling during transport, and one (1/21; 4.76%) balance problems during transport. Fear and anxiety were mainly noticed at loading (3/7; 42.86%), and during transport (2/7; 28.57%). Two respondents did not describe when fear and anxiety were noticed. 

#### 3.2.3. Descriptive Statistics of the Horse Injuries

Seventeen respondents reported that at least one of their horses suffered from a transport-related injury (17/148; 11.49%) (Table S5). Ten were mares (10/17; 58.82%), five were geldings (5/17; 29.41%), and one was a stallion (1/17; 5.88%). Eight respondents (8/17; 47.06%) indicated that the horses suffered a slight injury or bruises, three (3/17; 17.65%) deep wounds, two (2/17; 11.76%) bruises and hematomas, two (2/17; 11.76%) deep wounds and hematomas, and one (1/17; 5.88%) only hematomas. Eight horses (8/17; 47.06%) showed injuries in several anatomical locations, mainly consisting of back, hind legs, and tail wounds, and hematomas. The remaining eight (8/17; 47.06%) were injured on the head (2/17; 11.76%), front legs (1/17; 5.88%), back (1/17; 5.88%), hind legs (3/17; 17.65%), and on the tail (1/17; 5.88%). Seven respondents (7/17; 41.18%) reported that the injuries healed in one week, five in two weeks (5/17; 29.41%), two in one month (2/17; 11.76%), one in two months (1/17; 5.88%), and one reported that the horse had not yet reached a full recovery (1/17; 5.88%). Eleven respondents reported that the injuries happened during the transport (11/17; 64.71%), three at unloading (3/17; 17.65%), one at pre-loading (1/17; 5.88%), and one at loading (1/17; 5.88%). One respondent (1/17; 5.88%) did not provide any information about the injury sustained by the transported horse. 

Four out of the 17 (4/17; 23.53%) respondents reporting horse injuries declared that they also injured themselves during the same accident.

#### 3.2.4. Descriptive Statistics of the Horse Handler Injuries

Ten respondents reported they were injured during transport (10/140; 7.14%) (Table S5). Among them, four reported they were injured simultaneously with their horse (4/10; 40.00%). Four (4/10; 40.00%) were injured at loading, three (3/10; 30.00%) during transport, one (1/10; 10.00%) at unloading, and one (1/10; 10.00%) at pre-loading. One respondent (1/10; 10.00%) did not provide this information. Six respondents (6/10; 60.00%) reported severe wounds, fractures, and crush injuries in different body locations (head, hands, arms, legs, chest, and stomach) and reported that they needed hospital medications and medical assistance. One horse handler (1/10; 10.00%) declared he had a rope burn on his hands, which got better following self-medication; three respondents (3/10; 30.00%) did not answer. Five horse handlers (5/10; 50.00%) reported a median recovery time of one month (ranging from one week to six months).

### 3.3. Univariable and Stepwise Multivariable Logistic Regression for TRPBs

The Wald test *p*-values of all the predictive variables tested with the univariable logistic regression models for TRPBs are reported in Table S6. Predictive variables showing a Wald Test *p*-value < 0.05 are reported in Table 7.

In the univariable logistic regression, the use of sedatives or loading equipment predicted TRPBs, specifically the whip and other aids. The probability of displaying TRPBs increased by five times if the horse handlers reported sedating the horse before traveling, by seven and nine times, respectively, if the horse handler used a whip or other aids at loading, and by two times if any loading equipment was used.

The stepwise multivariable logistic regression model found that TRPBs were related to the gender of the horse handler, the lack of checking the brakes, the use of a whip, sedation of the horse, the vehicle design protection features, and using shavings as bedding (AIC = 94.05). The probability of displaying TRPBs increased by five times if the horse handler was female, by more than three times if the horse handler did not check brakes before transport, by more than five times if the horse handler used a whip at loading, by 13 times if the animal was sedated, and by five times if the vehicle did not have design features for horse protection and the bedding did not consist of shavings (Table 8). For this model, 134 observations were retained as complete for all the included variables.

### 3.4. Univariable and Stepwise Multivariable Logistic Regression for Horse Injury

The Wald test *p*-values of all the predictive variables tested with the univariable logistic regression models for transport-related horse injuries are reported in Table S7. The predictive variables showing a Wald Test *p*-value < 0.05 are reported in Table 9. The probability of horse injuries increased by almost three times if the horse handler did not check the brakes before transport, and by almost eight times if the animals showed TRPBs. 

The stepwise multivariable logistic regression model found that transport-related injuries in horses were associated with a lack of checking the brakes before transport, the presence of padding on the chest bar, the absence of a rubber mat as bedding, and the presence of TRPBs (AIC = 89.44). The probability of a horse sustaining a travel-related injury increased by three times if the horse handler did not check brakes before transport and if padding on the chest bar was present, by two times if a rubber mat was not present, and by eight times if the horse had TRPBs (Table 10). For this model, 137 observations were retained as complete for all the included variables.

## 4. Discussion

This research aimed to find, through a specific online survey, the frequency of TRBPs and injuries associated with horse transportation in Italy. TRPBs and horse injuries were reported by 14.45% and 11.49% of respondents, respectively, and association with factors that may increase or decrease their odds of occurring were identified. Although the collected data were insufficient to develop a truly representative picture of the Italian situation (due to insufficient sample size), our data indicate the significant impact that road transport has on the health and welfare of the horses and their handlers.

Road transport threatens the health and welfare of horses in Italy due to TRPBs and subsequent injuries. However, it is important to underline that TRPBs and transport injuries reported in this study were less frequent compared to rates described in previous publications [3,6,8,9,31]. The reason behind those low percentages may be due to respondents’ involvement in the equine industry. It is worth noting that half of our respondents were between 18 and 30 years old, and the majority of them were involved in the equine industry as amateurs for recreational purposes. It is well known that amateurs are less likely to report transport-related issues than professionals [8,9,10]. Amateurs also travel for shorter distances than professionals and move their horses rarely. In the current survey, ~43% moved their horses less than once in a month and 25% monthly for short distances only. Most respondents were female (77%). However, a connection between females and the occurrence of TRPBs may reflect a greater willingness than men to respond to surveys of this type, rather than indicating a true risk factor [6,40,41,42], or men may not be as aware of behavioral issues as women. Overall, considering that in Italy 70% of the practicing FISE members are women, the gender distribution of respondents was congruent with the distribution of gender within the equine Italian industry. 

Fourteen percent of the respondents reported having at least one horse with TRPBs, such as fear and anxiety preloading, refusal to get on the trailer, kicks, and loss of balance during transportation. In previous research, the percentage of respondents with horses with TRPBs was higher, between 55.6% [10] and 75% [8]. TRPBs may be reduced, at least in part, by applying appropriate training methods, such as habituation and self-loading as procedures [9,18,20,27,43,44]. In contrast, training methods involving the use of negative reinforcement or positive punishment were considered the most hazardous [20,31]. In our survey, most participants declared that their horses were trained to load and travel through self-loading and positive reinforcement or using a combination of negative reinforcement and positive punishment, however, no association between the training method and the presence of TRPBs was found, probably due to the small sample sizes in some categories, or it could be that even some techniques were not correctly applied. For instance, self-loading could be still coercive when horses are bullied/rushed by the handler just by body language or shouting. Our data showed a relationship between TRPBs and the use of loading equipment. The discovery of this finding agreed with the literature [22], confirming that the use of the whip and other equipment can increase the risk of a TRPB occurring. If a horse refuses to get on, the use of the whip as a positive punishment often fails. Whipping as a positive punishment was described by Houpt in 1982 as a temporary solution to the problem (the horse gets on in order to avoid being whipped) creating a negative association (loading into a trailer instills fear of being whipped) [11]. This consequently leads to TRPBs during loading onto a trailer [9]. The use of the whip can also increase the risk of TRPBs during transportation because a horse loaded with a whip is more scared and anxious. Consequently, the use of loading equipment such as a whip should not be recommended, and the application of the least traumatizing methods to train horses, such as habituation and self-encouraging [27,44,45], should be encouraged.

In our research, the presence of TRPB was positively associated with the use of a sedative during the pre-loading procedure (i.e., the use of sedative increased up to 13 times the possibility of reporting a horse with TRPB). Sedatives may be used with the assumption that sedated horses are more manageable and that this could be a good strategy to load them or because horses had TRPBs and the owners relied on sedation for those behavioral problems. Although sedation may be used to simplify the loading of the animal, it is well known that it can reduce the ability of the horse to balance [10]. Tranquilizers can affect the psychophysical condition of the horse, from body temperature to the ability to react. However, it is worth highlighting that sedated horses do not lose their ability to kick—rather, they overreact to stimuli, kicking even more than when they are not sedated; this may also explain the association we found between sedation and TRPBs [27,43]. For these reasons, to minimize TRPBs it is always recommended to train with the least traumatizing techniques mentioned above [12,20,27,32,44] instead of relying on the administration of sedatives.

TRPBs were also associated with vehicle features designed for protection of the horse and lack of shavings on the floor of the vehicle. Our results suggest that traveling without protective vehicle features and using shavings as bedding increased the exhibition of TRPBs up to five times. The use of protective design features in the vehicle, rather than on the horse, was positive and considered evidence of a desire for comfort for the animal [14]. Eighty-one percent of our respondents used protective equipment directly worn on their horses during transportation (leg bandages, tail guard/bandage, body rug, leg boots). It is believed by many that such equipment can prevent injuries to the horse during loading or transportation, but this is not confirmed by the literature. On the contrary, there is a connection between the usage of these types of transport protective equipment and the occurrence of TRPB [46]. Habituation to this type of equipment by the horse is necessary, because it may limit its movements, generating distress and TRPB. The usage of vehicles with features designed for protection during transport is, therefore, the best and simplest solution to prevent animals from being injured. The use of bedding is considered important because in transit animals produce a high quantity of urine, leading to unhealthy and slippery environments [22]. A slippery substrate interferes with balance and increases risk of falling. Considering that shavings have a significant absorbent potential, it also is also recommended to minimize TRPBs. 

The lack of control of the brakes of the trailer before the journey resulted in an association with both TRPBs and injuries. It was suggested that the horse’s ability to maintain balance in transit can be influenced by the mechanical condition of the vehicle, in particular brakes and suspension [22,46]. Improper braking can cause a loss of balance in the animal, which can be propelled forward inside the trailer with consequent injuries to the front of the head. Moreover, a horse that has previously lost its balance in transport starts to associate transport with a stressful moment, and become full of anxiety and fear. Stress connected to these emotions automatically leads to TRPB, and consequently to injuries [27]. An appropriate vehicle checklist was suggested as the best practice [8,46] to reduce both TRPBs and injuries, since accidents are often associated with a malfunction of the vehicle [6].

Injuries were associated with TRPBs and the presence of padding on the chest bar. Although the usage of protective design features inside the vehicle is a very effective method to limit accidents, our data stated that using padding on the chest bar increased by three times the probability for the horses to be injured. This could be associated with previous injuries that led the owners to put padding on the chest bar, the movement of the vehicle with the ability of the drivers, or an inappropriate position of the chest bar. Inappropriate driving maneuvers can also lead to a loss of balance in the animal, swinging forwards and backward and bumping into the chest bar. Therefore, the problem may not be with the bar padding, but rather the chest bar itself. Different containment systems (chest bar/bum bar) used to confine the horse inside the trailer or to minimize its movement may entrap the animal. For these reasons, protection bars for horses should only be marketed if “instantly removable” even with the horse’s bodyweight upon them. This recommendation avoids trapping injuries and helps rescuers [46]. Our research revealed a tendency between the lack of a rubber mat as flooring and injuries. As described in the literature, the use of a rubber mat is recommended because it helps the horse to cushion and prevent the penetration of the hoof through the floor of the vehicle in case of an accident [46]. The rubber mat provides a non-slip surface and may be useful to minimize transport-related injuries.

Overall, the strongest positive association was found between injuries and TRPBs. This was expected and agreed with the published literature [47]. During loading horses can express anxiety through rearing, kicking, pulling back, turning to the side, and biting others during the journey [19,21,24], with the risk of getting injured and hurting the other animals [3,27]. Thus, all the practices suggested above to reduce the occurrence of TRPBs are also useful for decreasing transport injuries.

Our research confirmed that transport is a risk also for horse handlers. Half of the participants were injured together with their horses. The majority of these Italian respondents declared they were injured during the loading or during the trip, suffering rope burns, head injuries, crush injuries, tendon and muscular injuries, soft tissue injuries, dislocations, and sprains. The body parts most exposed to injuries were the chest, the back, the legs, and the hands. Five of the seven people with back injuries went to first aid for medical care, whereas the others asked for medical assistance or self-medicated. Recovery from injury occurred with a healing period of between one week and six months. Our data are in line with the literature [7]. Despite the human effort in training horses to be desensitized from external stimuli, horses can often exhibit unpredictable behaviors; when they feel under threat they can run away, bite, kick, or crush, and cause injuries in humans [48]. Transportation can be considered a dangerous practice for people and animals. For this reason, it is important to have a good knowledge of health and workplace safety laws. Analyzing our data, it was found that 28% of the participants had a medium knowledge of the law and 25% had high knowledge. This could have influenced the low number of transport injuries observed in the survey. Good transport practices should be widely shared to improve the health not only of horses but also their handlers.

Our results have to be interpreted with caution because of some potential limitations, one of them being the prejudices of the respondents to the surveys [49]. Secondly, the demographic of the participants was a limitation, since this survey was distributed only on the web and promoted through social media. The survey was exclusively available online, so the data collected was elicited from people with access to the Internet. Therefore, generalization of the results is challenging [50]. In this survey, the number of participants did not reached the minimum target population needed in order to have a confidence level of 95% and an error rate of ±5%. Therefore, our data cannot be used to provide an exact estimate of the number of accidents that occurred and the factors increasing or decreasing their occurrence in Italy. Finally, Dean [51] identified non-response bias, recall bias, and social acceptability bias as factors that may confound the interpretation of survey data, and all may apply to this study. Despite these limitations, these findings represent a first attempt to indicate the ratio of accidents related to horse transportation in the Italian equine industry. The results should encourage the conduct of prospective and intervention studies to further investigate the associations found in the present study and to educate horse people on the need for standards for trailers and on best practices that can minimize the occurrence of TRPBs and injuries in both horses and handlers.

## 5. Conclusions

This online survey investigated for the first time the occurrence of TRPBs and transport injuries in the Italian equine industry, confirming that there were associations among those events and respondents’ transport practices. Although the responses did not reach the significant sample size desired, this is the first study reporting the standard practices of amateurs and professionals in the Italian horse industry and the effect of those practices on horse and human welfare. Our findings were in line with the literature, confirming that TRPBs may be a risk for both horses and handlers. Further prospective studies are needed to investigate how to train and manage horses for transport to reduce TRBPs and their important influence on the risk of transport-related injury.

## Figures and Tables

**Table 1:** Distribution pathways for the survey on horse road transport practices, transport-related problem behaviors, horse injuries, and horse handler injuries in Italy.

Category	Name	Method
Groups and Facebook pages	Performance Sales International (PSI) Horses Only Horse Lovers Equestrian Tourism Oristano is Your Sartiglia Horse Touring Horses Rome Pony Club Fieracavalli Verona Acavallo	Sharing the survey on Facebook pages and groups
Horse magazine	*The Portal of the Horse* *Cavallo Magazine* *Equestrian Insights*	Sharing the survey on their web and Facebook pages
Facebook groups of carriers	Live animal transport Cattle transport drivers Cattle truck Purchasing and selling trailers and horse transport vans	Sharing the survey in groups
Personal contacts	Official veterinarians Farmers/horse owners	Sharing the survey by e-mail

**Table 2:** Name and description of the candidate explanatory variables evaluated in a survey on horse road transport practices, transport-related problem behaviors, horse injuries, and horse handler injuries in Italy.

Name	Description	Categories
Respondent Details		
Gender		Female, Male, Other
Age		18–30, 31–45, 46+
Origin	The geographic region of Italy where the respondents were living.	Center, North, South
Involvement with the Equine Industry		
Sector	In which sector of the horse industry were they involved?	Equestrian sports (ES; endurance, dressage, show jumping), recreational riding (RR), Other (racing, breeding), Western (W)
Membership	Was the respondent a member of a horse-related association?	Yes/No
FISE (Italian Equestrian Sports Federation)	Was the respondent associated with the Italian Equestrian Sports Federation (FISE)?	Yes/No
Involvement	Nature of the respondent’s involvement with horses.	Amateur (primarily involved with horses as a hobby), Professional (primarily involved with horses for financial reward)
Experience/Knowledge of the Respondent		
Experience	Respondent’s years of experience in handling horses.	0–10, 11–20, 21–30, 31+
Qualification	Possession of one or more equine industry qualifications.	Yes/No
		
Driving license	Respondent’s class of driving license.	None, B (car), C (rigid-heavy vehicle), CE (articulate-heavy vehicle)
Knowledge of health and safety at work law	Respondent’s self-assessment of their understanding of the health and safety work law (legislative decree 81/2008).	1—low, 2—some, 3—moderate, 4—high
AWC (Animal Welfare Code)	Respondent’s self-assessment of their understanding of CE regulation nr. 1/2005—protection of animals during transport and related operations.	1—low, 2—some, 3—moderate, 4—high
Distress	Respondent’s self-assessment of their ability to identify a horse in distress.	1—low, 2—some, 3—moderate, 4—high
Horse and Journey Details		
Number of horses owned	How many horses does the respondent own?	<8, 8–24, 25–39, ≥40
Frequency of transport	Frequency of organized transport events.	Less than once a month, Fortnightly, Weekly, Monthly
Journey distance	Average journey distance (km).	0–39, 40–69, 70–149, ≥150
FFT (frequency fitness for travel)	Frequency of assessment of fitness for travel before moving horses.	1–2 never and sometimes, 3—about half the time
Pre-Transport Practices		
Mechanical checklist	Frequency of use of a mechanical checklist on the transport vehicle before moving horses.	1—never, 2—sometimes, 3—about half the time
Brakes	The respondent checks the brakes.	Yes/No
Lights	The respondent checks the lights.	Yes/No
Tire pressure	The respondent checks the tire pressure.	Yes/No
Wheel nuts	The respondent checks the wheel nuts.	Yes/No
Hydraulic fluid levels	The respondent checks the hydraulic fluid levels.	Yes/No
Sides/walls	The respondent checks the sides/walls.	Yes/No
Floor	The respondent checks the floor.	Yes/No
Towbar attachment	The respondent checks the towbar attachment.	Yes/No
Windows	The respondent checks the windows.	Yes/No
Ventilation	The respondent checks the ventilation.	Yes/No

Sedation	Use of sedatives or other products to calm the horse before transport.	Yes/No
Transport Protections and Horse Training for Transport		
Total protection	Use of horse protective equipment while traveling.	0, 1, ≥2
Leg bandages	The respondent uses leg bandages.	Yes/No
Tail guard/bandage	The respondent uses a tail guard/bandage.	Yes/No
Body rugs	The respondent uses a body rug.	Yes/No
Leg boots	The respondent uses leg boots.	Yes/No
Horse training	Did the respondents train their horses for loading and traveling?	Yes/No
Type of training	What kind of help do you use to load the horse?	Habituation (H), not specified (NS), no training applied (NT), positive reinforcement and self-loading (R+SL), the combination of negative reinforcement and positive punishment (R-P+)
Loading Practices		
Loading equipment	Do you use any equipment or aids for loading your horse?	Yes/No
Total equipment	How many aids (apart from a halter) do you use for loading?	0, 1, ≥2
Whip	The respondent uses a whip as a loading aid.	Yes/No
Food for loading	The respondent uses food as a loading aid.	Yes/No
Bum rope	The respondent uses a bum rope as a loading aid.	Yes/No
Other aids	The respondent uses other aids for loading (i.e., bridle, stallion bit, load ‘n’ tie)	Yes/No
Vehicle Design and Transport Practices		
Containment in the vehicle	How was the horse restrained inside the vehicle?	Cross tie, Tie up on a short rope (less than 30 cm), Tie up on a long rope (more than 30 cm), No containment
Vehicle protections	Use of any protective modifications within the vehicle during transport?	Yes/No
Padding on partitions	The respondent uses padding on partitions for protection.	Yes/No
Padding on bum bar/behind horse	The respondent uses padding on the bum bar/behind the horse for protection.	Yes/No
Padding on chest bar	The respondent uses padding on the chest bar for protection.	Yes/No
Partition extended to floor	The respondent uses a partition extended to the floor for protection.	Yes/No
Food en route	Feeding when traveling.	Yes/No
Straw	The respondent uses straw as bedding on the floor.	Yes/No
Shavings	The respondent uses shavings as bedding on the floor.	Yes/No
Rubber mat	The respondent uses a rubber mat as bedding on the floor.	Yes/No
Sawdust	The respondent uses sawdust as bedding on the floor.	Yes/No
Behavioral Problem		
TRPBs (transport-related behavioral problems)	Whether the horse showed one or more transport-related behavioral problems.	Yes/No

**Table 3:** Frequency table of the replies (*n* = 148) to the experience/knowledge of the respondent in a survey on horse road transport practices, transport-related problem behaviors, horse injuries, and horse handler injuries in Italy.

Experience/Knowledge of the Respondent			
Variable Name	Category	Count	Percentage
Experience (years)	0–10	40	28.57
	11–20	55	39.29
	21–30	33	23.57
	31+	12	8.57
	Total	140	100
	Missing values	8	5.41
Qualification	No	56	37.84
	Yes	92	62.16
	Total	148	100
Driving license	No	15	10.14
	B (car)	95	64.19
	C (rigid-heavy vehicle)	20	13.51
	CE (articulate-heavy vehicle)	18	12.16
	Total	148	100
Knowledge of health and safety at work act	1 (low)	33	22.60
	2 (some)	34	23.29
	3 (moderate)	42	28.77
	4 (high)	37	25.34
	Total	146	100
	Missing values	2	1.35
Knowledge of Animal Welfare Code (AWC)	1 (low)	42	28.77
	2 (some)	31	21.23
	3 (moderate)	30	20.55
	4 (high)	43	29.45
	Total	146	100
	Missing values	2	1.35
Distress	1 (low)	9	6.12
	2 (some)	24	16.33
	3 (moderate)	67	45.58
	4 (high)	47	31.97
	Total	147	100
	Missing values	1	0.68

**Table 4:** Frequency table of the replies (*n* = 148) to the horse and journey details in a survey on horse road transport practices, transport-related problem behaviors, horse injuries, and horse handler injuries in Italy.

Horse and Journey Details			
Variable Name	Category	Count	Percentage
Number of horses owned	<8	34	23.61
	8–24	34	23.61
	25–39	30	20.83
	≥40	46	31.94
	Total	144	100
	Missing values	4	2.70
Frequency of transport	Weekly	16	10.81
	Fortnightly	32	21.62
	Monthly	37	25.00
	Less than once a month	63	42.57
	Total	148	100
Journey distance (kilometers)	0–39	36	25.17
	40–69	31	21.68
	70–149	35	24.48
	≥150	41	28.67
	Total	143	100
	Missing values	5	3.38
Checked for fitness for transport	1–2 (never and sometimes)	27	18.24
	3 (about half the time)	121	81.76
	Total	148	100

**Table 5:** Frequency table of the replies (*n* = 148) to the pre-transport practices in a survey on horse road transport practices, transport-related problem behaviors, horse injuries, and horse handler injuries in Italy.

	Pre-Transport Practices		
Variable Name	Category	Count	Percentage
Mechanical checklist	1 (never)	43	30.28
	2 (sometimes)	53	37.32
	3 (about half the time)	46	32.39
	Total	142	100
	Missing values	6	4.05
Brakes	No	47	33.57
	Yes	93	66.43
	Total	140	100
	Missing values	8	5.41
Lights	No	45	32.14
	Yes	95	67.86
	Total	140	100
	Missing values	8	5.41
Tire pressure	No	38	27.14
	Yes	102	72.86
	Total	140	100
	Missing values	8	5.41
Wheel nuts	No	106	75.71
	Yes	34	24.29
	Total	140	100
	Missing values	8	5.41
Hydraulic fluid levels	No	94	67.14
	Yes	46	32.86
	Total	140	100
	Missing values	8	5.41
Sides/walls	No	83	59.29
	Yes	57	40.71
	Total	140	100
	Missing values	8	5.41
Floor	No	72	51.80
	Yes	67	48.20
	Total	139	100
	Missing values	9	6.08
Towbar attachment	No	53	37.86
	Yes	87	62.14
	Total	140	100
	Missing values	8	5.41
Windows	No	88	62.86
	Yes	52	37.14
	Total	140	100
	Missing values	8	5.41
Ventilation	No	66	47.14
	Yes	74	52.86
	Total	140	100
	Missing values	8	5.41
Sedation	No	129	88.36
	Yes	17	11.64
	Total	146	100
	Missing values	2	1.35

**Table 6:** Frequency table of the replies (*n* = 148) to loading practices in a survey on horse road transport practices, transport-related problem behaviors, horse injuries, and horse handler injuries in Italy.

Loading Practices			
Variable Name	Category	Count	Percentage
Loading equipment	No	105	70.95
	Yes	43	29.05
	Total	148	100
Total equipment	0	104	70.74
	1	23	15.64
	≥2	20	13.60
	Total	147	100
	Missing values	1	0.68
Whip	No	137	93.20
	Yes	10	6.80
	Total	147	100
	Missing values	1	0.68
Food for loading	No	114	77.55
	Yes	33	22.45
	Total	147	100
	Missing values	1	0.68
Bum rope	No	129	87.76
	Yes	18	12.24
	Total	147	100
	Missing values	1	0.68
Other aids	No	138	93.88
	Yes	9	6.12
	Total	147	100
	Missing values	1	0.68

**Table 7:** Results of the univariable logistic regression with the variables associated with transport-related problem behaviors (TRPBs) reported in the replies (*n* = 148) to a survey on horse road transport in Italy. Results are expressed as the odds ratio (OR), confidence interval (95% CI), *p*-value for each predictive variable and total *p* value of the Wald test.

Variable	Category	OR	CI 5–95%	*p*-Value	Wald Test
Sedation	No	Ref			0.003
	Yes	5.65	1.80–17.26	0.002	
Whip	No	Ref			0.004
	Yes	7.44	1.88–29.62	0.003	
Other aids	No	Ref			0.002
	Yes	9.36	2.26–41.48	0.001	
Loading equipment	No	Ref			0.049
	Yes	2.61	1.00–6.78	0.046	

**Table 8:** Results of the stepwise multivariable logistic regression analysis with the variables associated with transport-related problem behaviors (TRPBs) reported in the replies (*n* = 148) to a survey on horse road transport in Italy. Results are expressed as the odds ratio (OR), confidence interval (95% CI), *p*-value for each predictive variable and total *p* value of the Wald test.

Variable	Category	Proportion (%)	OR	CI 5–95%	*p*-Value	Wald Test
Respondent gender	Male	32/134 (23.88)	Ref			0.034
	Female	102/134 (76.12)	5.72	1.12–49.00	0.063	
Brakes	Yes	88/134 (65.67)	Ref			0.040
	No	46/134 (34.33)	3.46	1.06–12.32	0.044	
Whip	No	125/134 (93.28)	Ref			0.064
	Yes	9/134 (6.72)	5.36	0.90–34.08	0.063	
Sedation	No	118/134 (88.06)	Ref			0.002
	Yes	16/134 (11.94)	13.48	2.64–82.87	0.002	
Vehicle design protections	Yes	122/134 (91.04)	Ref			0.050
	No	12/134 (8.96)	5.08	1.00–24.60	0.041	
Shavings	Yes	83/134 (61.94)	Ref			0.049
	No	51/134 (38.06)	4.93	1.32–22.84	0.025	

**Table 9:** Results of the univariable logistic regression with the variables associated with transport-related horse injuries reported in the replies (*n* = 148) to a survey on horse road transport in Italy. Results are expressed as the odds ratio (OR), confidence interval (95% CI), *p*-value for each predictive variable and total *p* value of the Wald test.

Variable	Category	OR	CI 5–95%	*p*-Value	Wald Test
Brakes	Yes	Ref			0.050
	No	2.91	1.01–8.70	0.048	
TRPBs	No	Ref			0.0004
	Yes	7.86	2.56–24.32	0.0003	

**Table 10:** Results of the stepwise multivariable logistic regression analysis with variables associated with the occurrence of transport-related horse injuries reported in the replies (*n* = 148) to a survey on horse road transport in Italy. Results are expressed as the odds ratio (OR), confidence interval (95% CI), *p*-value for each predictive variable and total *p* value of the Wald test.

Variable	Category	Proportion (%)	OR	CI 5–95%	*p*-Value	Wald Test
Brakes	Yes	90/137 (65.69)	Ref			0.043
	No	47/137 (34.31)	3.48	1.04–12.55	0.046	
Padding on chest bar	No	74/137 (54.01)	Ref			0.038
	Yes	63/137 (45.99)	3.63	1.07–14.09	0.046	
Rubber mat	Yes	83/137 (60.58)	Ref			0.149
	No	54/137 (39.42)	2.34	0.74–7.89	0.153	
TRPBs	No	118/137 (86.13)	Ref			0.001
	Yes	19/137 (17.87)	8.22	2.34–30.34	0.001	

## Data Availability

Data available on request due to restrictions (ethical approval).

## Supplementary Materials

The following are available online at https://www.mdpi.com/2076-2615/11/1/223/s1, Survey. Table S1. Frequency table of the replies (*n* = 148) to respondents details related questions in a survey on horse road transport and the related behavioral problems, horse injuries and horse handler injuries in Italy. Table S2. Frequency table of the replies (*n* = 148) to the involvement with the equine industry in a survey on horse road transport and the related behavioral problems, horse injuries and horse handler injuries in Italy. Table S3. Frequency table of the replies (*n* = 148) to the transport protections and horse training for transport in a survey on horse road transport and the related behavioral problems, horse injuries and horse handler injuries in Italy. Table S4. Frequency table of the replies (*n* = 148) to the vehicle design and the transport practices in a survey on horse road transport and the related behavioral problems, horse injuries and horse handler injuries in Italy. Table S5. Frequency table of the replies (*n* = 148) in a survey on horse road transport and the related behavioral problems (TRPBs), horse injuries and horse handler injuries in Italy. Table S6: Wald test *p*-values of the univariate logistic regressions for the predictive variables associated with the transport-related behavioral problems (TRPBs) reported in the replies (*n* = 148) to a survey on horse road transport in Italy. Table S7: Wald test *p*-values of the univariate logistic regressions for the predictive variables associated with the transport-related horse injuries reported in the replies (*n* = 148) to a survey on horse road transport in Italy.
