# Supplementary material for: Equine Transport-Related Problem Behaviors and Injuries: A Survey of Italian Horse Industry Members
Source: Animals (Basel). 2021 Jan 18;11(1):223. doi: 10.3390/ani11010223 (PMC7831101; doi:10.3390/ani11010223)
Supplement: Supplementary file 1 [file animals-11-00223-s001.zip › supplementary/Suplementary Tables Dai et al. submitted R1 1301.docx]

**Table S1**. Frequency table of the replies (n=148) to respondents details related questions in a survey on horse road transport and the related behavioral problems, horse injuries and horse handler injuries in Italy.

| **Respondents details** | | | |
| --- | --- | --- | --- |
| **Variable Name** | **Category** | **Count** | **Percentage** |
| Respondent’s gender | Female | 111 | 77.08 |
|  | Male | 33 | 22.92 |
|  | Total | 144 | 100 |
|  | Other/Missing | 4 | 2.78 |
| Age | 18-30 | 73 | 50.34 |
|  | 31-45 | 41 | 28.28 |
|  | 46+ | 31 | 21.38 |
|  | Total | 145 | 100 |
|  | Missing Values | 3 | 2.03 |
| Origin | Center | 20 | 15.87 |
|  | North | 91 | 72.22 |
|  | South | 15 | 11.90 |
|  | Total | 126 | 100 |
|  | Missing Values | 22 | 14.86 |

**Table S2**. Frequency table of the replies (n=148) to the involvement with the equine industry in a survey on horse road transport and the related behavioral problems, horse injuries and horse handler injuries in Italy.

| **Involvement with the equine industry** | | | |
| --- | --- | --- | --- |
| **Variable Name** | **Category** | **Count** | **Percentage** |
| Sector | Recreational Riding (RR) | 35 | 23.64 |
|  | Equestrian Sport (ES) | 77 | 52.03 |
|  | Other | 17 | 11.49 |
|  | Western (W) | 19 | 12.84 |
|  | Total | 148 | 100 |
| Membership | No | 26 | 17.57 |
|  | Yes | 122 | 82.43 |
|  | Total | 148 | 100 |
| Italian Equestrian Sports Federation (FISE) | No | 59 | 39.86 |
|  | Yes | 89 | 60.14 |
|  | Total | 148 | 100 |
| Involvement | Amateur | 97 | 65.99 |
|  | Professional | 50 | 34.01 |
|  | Total | 147 | 100 |
|  | Missing Values | 1 | 0.68 |

**Table S3**. Frequency table of the replies (n=148) to the transport protections and horse training for transport in a survey on horse road transport and the related behavioral problems, horse injuries and horse handler injuries in Italy.

| **Transport protections and horse training for transport** | | | |
| --- | --- | --- | --- |
| **Variable Name** | **Category** | **Count** | **Percentage** |
| Total protection | No | 27 | 18.49 |
|  | 1 | 27 | 18.49 |
|  | 2 | 49 | 33.56 |
|  | 3 | 30 | 20.55 |
|  | 4+ | 13 | 8.90 |
|  | Total | 146 | 100 |
|  | Missing Values | 2 | 1.35 |
| Leg bandages | No | 52 | 35.62 |
|  | Yes | 94 | 64.38 |
|  | Total | 146 | 100 |
|  | Missing Values | 2 | 1.35 |
| Tail guard/bandage | No | 64 | 43.84 |
|  | Yes | 82 | 56.16 |
|  | Total | 146 | 100 |
|  | Missing Values | 2 | 1.35 |
| Body rug | No | 86 | 58.90 |
|  | Yes | 60 | 41.10 |
|  | Total | 146 | 100 |
|  | Missing Values | 2 | 1.35 |
| Leg boots | No | 115 | 78.77 |
|  | Yes | 31 | 21.23 |
|  | Total | 146 | 100 |
|  | Missing Values | 2 | 1.35 |
| Horse Training | No | 56 | 37.84 |
|  | Yes | 92 | 62.16 |
|  | Total | 148 | 100 |
| Type of Training | Habituation (H) | 25 | 16.89 |
|  | Not specified (NS) | 34 | 22.97 |
|  | No training (NT) | 56 | 37.84 |
|  | Positive reinforcement and self-loading (R+SL) | 15 | 10.14 |
|  | Negative reinforcement and positive punishment (R-P+) | 18 | 12.16 |
|  | Total | 148 | 100 |

**Table S4**. Frequency table of the replies (n=148) to the vehicle design and the transport practices in a survey on horse road transport and the related behavioral problems, horse injuries and horse handler injuries in Italy.

| **Vehicle design and transport practices** | | | |
| --- | --- | --- | --- |
| **Variable Name** | **Category** | **Count** | **Percentage** |
| Containment in the vehicle | Cross tie | 75 | 51.02 |
|  | Tie up on a short rope (Less than 30 cm) | 33 | 22.45 |
|  | Tie up on a long rope (More than 30 cm) | 29 | 19.73 |
|  | No containment | 10 | 6.80 |
|  | Total | 147 | 100 |
|  | Missing Values | 1 | 0.68 |
| Vehicle protections | No | 15 | 10.14 |
|  | Yes | 133 | 89.86 |
|  | Total | 148 | 100 |
| Padding on partitions | No | 51 | 34.46 |
|  | Yes | 97 | 65.54 |
|  | Total | 148 | 100 |
| Padding on bum bar/behind horse | No | 63 | 42.57 |
|  | Yes | 85 | 57.43 |
|  | Total | 148 | 100 |
| Padding on chest bar | No | 81 | 54.73 |
|  | Yes | 67 | 45.27 |
|  | Total | 148 | 100 |
| Partition extended to floor | No | 119 | 80.41 |
|  | Yes | 29 | 19.59 |
|  | Total | 148 | 100 |
| Food *en route* | No | 64 | 43.24 |
|  | Yes | 84 | 56.76 |
|  | Total | 148 | 100 |
| Straw | No | 125 | 85.03 |
|  | Yes | 22 | 14.97 |
|  | Total | 147 | 100 |
|  | Missing Values | 1 | 0.68 |
| Shavings | No | 61 | 41.50 |
|  | Yes | 86 | 58.50 |
|  | Total | 147 | 100 |
|  | Missing Values | 1 | 0.68 |
| Rubber mat | No | 58 | 39.73 |
|  | Yes | 88 | 60.27 |
|  | Total | 146 | 100 |
|  | Missing Values | 2 | 1.35 |
| Sawdust | No | 128 | 87.07 |
|  | Yes | 19 | 12.93 |
|  | Total | 147 | 100 |
|  | Missing Values | 1 | 0.68 |

**Table S5**. Frequency table of the replies (n=148) in a survey on horse road transport and the related behavioral problems (TRPBs), horse injuries and horse handler injuries in Italy.

| **TRPBs and injuries in horses and horse handlers** | | | |
| --- | --- | --- | --- |
| **Variable Name** | **Category** | **Count** | **Percentage** |
| TRPBs | No | 124 | 85.52 |
|  | Yes | 21 | 14.45 |
|  | Total | 145 | 100 |
|  | Missing Values | 3 | 2.03 |
| Horse transport injuries | No | 131 | 88.51 |
|  | Yes | 17 | 11.49 |
|  | Total | 148 | 100 |
| Horse handler injuries/accident | No | 130 | 92.86 |
|  | Yes | 10 | 7.14 |
|  | Total | 140 | 100 |
|  | Missing Values | 8 | 5.41 |

**Table S6**: Wald test *P*-values of the univariate logistic regressions for the predictive variables associated with the transport-related behavioral problems (TRPBs) reported in the replies (n=148) to a survey on horse road transport in Italy.

| **Variable** | **Wald test P-values** |
| --- | --- |
| *Respondents details* | |
| Gender | 0.146 |
| Age | 0.770 |
| Origin | 0.529 |
| *Involvement with the equine industry* | |
| Sector | 0.657 |
| Membership | 0.210 |
| Italian Equestrian Sports Federation (FISE) | 0.546 |
| Involvement | 0.137 |
| *Experience/knowledge of the respondent* | |
| Experience | 0.621 |
| Qualification | 0.344 |
| Driving license | 0.902 |
| Knowledge of health and safety work act | 0.085 |
| Animal welfare code (AWC) | 0.315 |
| Distress | 0.652 |
| *Horse and journey details* | |
| Number of horses | 0.557 |
| Frequency of transport | 0.609 |
| Journey distance | 0.379 |
| Frequency fitness for travel (FFT) | 0.957 |
| *Pre-transport practices* | |
| Mechanical checklist | 0.725 |
| Brakes | 0.195 |
| Lights | 0.531 |
| Tyre pressure | 0.294 |
| Wheel nuts | 0.160 |
| Hydraulics fluids level | 0.488 |
| Sides/Walls | 0.091 |
| Floor | 0.330 |
| Towbar attachment | 0.722 |
| Windows | 0.618 |
| Ventilation | 0.349 |
| Sedation | 0.003 |
| *Transport protections and horse training for transport* | |
| Total protection | 0.992 |
| Leg bandage | 0.277 |
| Tail guard/bandage | 0.649 |
| Body rug | 0.219 |
| Leg boots | 0.766 |
| Horse training | 0.292 |
| Type of training | 0.794 |
| *Loading practices* | |
| Aids | 0.049 |
| Total equipment | 0.095 |
| Whip | 0.004 |
| Food for loading | 0.440 |
| Bum rope | 0.666 |
| Other aids | 0.002 |
| *Vehicle design and transport practices* | |
| Containment in the vehicle | 0.136 |
| Vehicle protections | 0.129 |
| Padding on partitions | 0.347 |
| Padding on bum bar/behind horse | 0.628 |
| Padding on chest bar | 0.845 |
| Partition extended to floor | 0.262 |
| Food *en route* | 0.157 |
| Straw | 0.241 |
| Shavings | 0.105 |
| Rubber mat | 0.485 |
| Sawdust | 0.099 |

**Table S7**: Wald test *P*-values of the univariate logistic regressions for the predictive variables associated with the transport-related horse injuries reported in the replies (n=148) to a survey on horse road transport in Italy.

| **Variable** | **Wald test *P*-value** |
| --- | --- |
| *Respondents details* | |
| Gender | 0.259 |
| Age | 0.780 |
| Origin | 0.406 |
| *Involvement with the equine industry*   \| *Involvement with the equine industry* \| \| --- \| | |
| Sector | 0.281 |
| Membership | 0.210 |
| FISE | 0.061 |
| Involvement | 0.671 |
| *Experience/knowledge of the respondent* | |
| Experience | 0.926 |
| Qualification | 0.450 |
| Driving license | 0.615 |
| Knowledge of health and safety work act | 0.571 |
| AWC | 0.321 |
| Distress | 0.708 |
| *Horse and journey details* | |
| Number of horses | 0.619 |
| Frequency of transport | 0.664 |
| Journey distance | 0.990 |
| FFT | 0.214 |
| *Pre-transport practices* | |
| Mechanical checklist | 0.783 |
|  | 0.050 |
| Lights | 0.297 |
| Tyre pressure | 0.122 |
| Wheel nuts | 0.586 |
| Hydraulics fluids level | 0.214 |
| Sides/Walls | 0.781 |
| Floor | 0.879 |
| Towbar attachment | 0.607 |
| Windows | 0.294 |
| Ventilation | 0.200 |
| Sedation | 0.118 |
| *Transport protections and horse training for transport* | |
| Total protection | 0.742 |
| Leg bandage | 0.277 |
| Tail guard/bandage | 0.211 |
| Body rug | 0.607 |
| Leg boots | 0.386 |
| Horse training | 0.180 |
| Type of training | 0.927 |
| *Loading practices* | |
| Aids | 0.596 |
| Total equipment | 0.485 |
| Whip | 0.873 |
| Food for loading | 0.276 |
| Bum rope | 0.949 |
| Other aids | 0.317 |
| *Vehicle design and transport practices* | |
| Containment in the vehicle | 0.818 |
| Vehicle protections | 0.992 |
| Padding on partitions | 0.320 |
| Padding on bum bar/behind horse | 0.252 |
| Padding on chest bar | 0.240 |
| Partition extended to floor | 0.164 |
| Food *en route* | 0.395 |
| Straw | 0.300 |
| Shavings | 0.314 |
| Rubber mat | 0.243 |
| Sawdust | 0.880 |
| *Behavioral problems* | |
| TRPBs | 0.0004 |
