# Supplementary material for: Equine Transport-Related Problem Behaviors and Injuries: A Survey of Italian Horse Industry Members
Source: Animals (Basel). 2021 Jan 18;11(1):223. doi: 10.3390/ani11010223 (PMC7831101; doi:10.3390/ani11010223)
Supplement: Supplementary file 1 [file animals-11-00223-s001.zip › supplementary/Supplementary Survey Dai et al. survey def.doc]

**Survey**

Gender

Male

Female

Other

2.How old are you?_________(years)

3.What is the postcode of the place where the horse is located?

4.Which sector of the horse industry are you primarily involved in?

Thoroughbred racing

Harness Racing

Pony club

Endurance

Horse Breeding

Recreational riding

Dressage

Show Jumping

Cross country

Reining

Show

Polo

Slaughter

Other (specify)

5.Are you a member of any equestrian organization?

I am not a member of any equestrian organization

Italian federation of equestrian sports (FISE)

Italian Federation of Equestrian Tourism and Trekking (FITETREC)

National organization of equestrian environmental guide (ENGEA)

Equestrian training school (SEF Italia)

Thoroughbred racing/Standardbred racing (ex UNIRE)

Other (specify)

6.What is your relationship with the horse?

Professional (e.g. has a financial gain from horses)

Amateur (e.g. involved with horses as a hobby)

7.How many years of experience do you have with handling horses?______

8.Do you have any qualifications recognized by technical institutions? (more options are accepted)

FISE (specify)

ENGEA (specify)

Certificate of competence (according to 1/2005/CE regulation)

Licence for harness racing (driver/gentleman)

Jockey Club

Other (specify)

No, I do not have any qualifications for the equine industry

9.Approximately, How many horses do you have on your property?______

10.What type of driving licence do you hold? (more options are accepted)

None

Learner’s permit

B

C

CE

Other (please specify)

11.How do you rank your understanding of Health and Safety at Work Act (legislative decree of 9 April 2008 nr. 81)?

1 – low

2 – some

3 -moderate

4 – high

5 - very high

12.How do you rank your understanding of the transport legislation (CE regulation nr. 1/2005)?

1 – low

2 – some

3 -moderate

4 – high

5 - very high

13.How do you grade your ability in identifying a horse in distress?

1 – low

2 – some

3 – moderate

4 – high

5 - very high

14.How often do you transport your horse?

Daily

2 - 5 times a week

Weekly

Fortnightly

Monthly

Less than once a month

15.What is the average kilometers traveled of the last year due to horse transportation? __

16.How likely are you to assess the fitness of your horse for travel before transporting it?

1 – never

2 – sometimes

3 - about half the time

4 - most of the time

5 - always

17.How likely are you to follow a check list on the transportation vehicle before travelling?

1 – never

2 – sometimes

3 - about half the time

4 - most of the time

5 – always

18.What do you check on your transport vehicle before travelling? (tick all that is applicable)

None

Brakes

Lights

Tyre pressure

Wheel nuts

Hydraulic fluid levels

Sides/walls

Floor

TOWBAR ATTACHMENT

Windows

Ventilation

Other ____________________

19.Do you assess the fitness for travel before transporting your horse?

Yes

No

20.Do you use sedation or other product to calm your horse prior to transportation?

All the time

Often

Sometimes

Rarely

Never

21.Do you use protective equipment on your horse? (tick all that apply)

None

Leg boots

Leg bandages

Poll protector

Tail guard/bandage

Neck rug

Body rug

Other (please specify) ____________________

22.Have you specifically trained your horses for loading and traveling?

No

Yes

Please describe how you trained your horse for loading and traveling:_____

23.Do you use any equipment or aids for loading your horse? (more options are accepted)

None

Whip

Bum rope

Food

Halter and rope

Bridle

Stallion bit

Load 'n' tie

Other (please specify) ____________________

24.Do you restrain your horse in the transport vehicle?

No

Tie up on a short rope (less than 30 cm)

Tie up on a long rope (more than 30 cm)

Cross tie

Hobble

Other (please specify) ____________________

25.Is there any protection (i.e. padding) in the transport vehicle? (more options are accepted)

Padding on wall

Padding on chest bar

Padding on partition

Padding on bum bar/behind horse

Partition extended to floor

Stallion guard

Other (please specify) ____________________

26.Do you offer food when travelling?

Yes - grain/complete feed

Yes - hay

No

27.Do you use any form of bedding on the floor?

Straw

Sawdust

Shavings

Rubber mat

Nothing

28.Do your horses have behavioural problems linked to transportation?

(e.g. anxiety, flight response, fight response, kicking, rearing, refusal to load)

Yes (please describe)

No

29.In which phase have you noticed the behavioural problem?

Pre-loading (e.g. vocalisation, pawing, shaking)

Loading (e.g. refusal to load, rearing, pulling back, kicking, pawing)

Travelling (e.g. kicking, biting, scrambling, pawing)

Unloading (e.g. rushing off, freezing)

30.Has your horse had any injuries related to transport within the last 2 years?

Yes

No

**Injured horse**

31.When during the trip did the injury occur?

Pre-Loading

Loading

During the journey

Unloading

I don't know

32.What type of injury occurred?

Shallow cut or wound

Deep cut or wound

Fracture or broken bone

Bruise

Skin or tail rubbed raw

Other (please specify) ____________________

33.Which part of the horse got injured? PICTURE (multiple options are possible)

1-Head

2-Neck

3-Chest

4-Flanc

5-Front leg

6-Back leg

7-Tail

Other (please specify) ____________________

34.How long did it take for the horse to recover?

The horse did not recover and had to be put down

1 week

2 weeks

Up to 1 month

Up to 2 months

>2 months

The horse has not yet recovered

**Check if injury**

35.Have you experienced an injury to yourself while you were transporting a horse in the last 2 years?

No - leave the survey

Yes

**Injury to person**

36.Did you sustain this injury at the same time of the horse when traveling or during the related activities?

Yes, the same accident described in this survey.

Yes, the same accident to a horse not described in this survey.

no

37.When did you sustained the injury?

Pre-loading/handling

Loading

Travelling

Unloading

38.Do you want to provide more information about your injury?

No - leave the survey

Yes

39.What type of injury did you sustained and in which part of the body?

Head injury

Face injury

Injuries to the teeth

Fracture

Crush injuries

Internal Injuries

Lacerations or open wounds

Bruises

Displacement/Sprain

Muscle injury

Rope Burn

Other (please specify)____________

40.Where did you got injured?

1-Head or Face

2-Neck

3-Back

4-Arms

5-Hands

6-Pelvic bones

7-Feet

8-Chest

9-Belly

41.Which clinical treatment did you get for the injury?

None

Self-medication

First Aid

Medical assistance

Hospitalisation

Other (please specify)

42.Did you recover from the injury?

No

Yes (specify the necessary time for recovery)
